# Pollution Characteristics and Risk Prediction of Endocrine Disruptors in Lakes of Wuhan

**DOI:** 10.3390/toxics10020093

**Published:** 2022-02-18

**Authors:** Yurui Zhang, Jun Cao, Tan Ke, Yue Tao, Wanyin Wu, Panpan Wang, Min Zhou, Lanzhou Chen

**Affiliations:** 1 Hubei Key Laboratory of Biomass Resource Chemistry and Environmental Biotechnology, Hubei Research Center of Environment Remediation Technology, School of Resource & Environmental Sciences, Wuhan University, Wuhan 430079, China; 2017282050281@whu.edu.cn (Y.Z.); ketan@whu.edu.cn (T.K.); taoyue@whu.edu.cn (Y.T.); 2019202050072@whu.edu.cn (W.W.); wangpanpan@whu.edu.cn (P.W.); zhoumin@whu.edu.cn (M.Z.); 2 School of Chemistry and Environmental Engineering, Hanjiang Normal University, Shiyan 442000, China; hbchen7112@hubu.edu.cn

**Keywords:** endocrine-disrupting compounds (EDCs), lakes, occurrence, environmental risk assessment, redundancy analysis

## Abstract

As a new and ubiquitous trace organic pollutant, endocrine-disrupting compounds (EDCs) can cause endocrine-disrupting effects on organisms even at low levels. However, little information is available on the resource and assessment of EDC risks in the water environment. The study area was selected based on the paucity of information on the pollution status of inland lakes. Wuhan has numerous and diverse types of lakes which receive micropollutants from different pathways. In this study, the spatial distribution, occurrence, quantity and ecological risks of EDCs in 12 lakes were investigated. Five EDCs, including 17-alpha-ethinylestradiol (17α-EE_2_), estrone (E_1_), β-estradiol (β-E_2_), estriol (E_3_) and bisphenol A (BPA) were detected in surface waters. The distribution of EDC content in the lakes was ordered as follows: exurban zone < suburban area < urban areas. The pollution sources in remote lakes mainly included agricultural and aquaculture wastewater, while those in suburban and urban areas included domestic or industrial wastewater. Areas with higher EDC content were frequently related to agricultural activities, aquaculture water or dense populations. Water quality parameters, including dissolved oxygen, pH and water temperature, were significantly related to the occurrence and distribution of EDCs in the lakes. Risk assessment demonstrated that the occurrence of EDCs posed minimum to medium risk to aquatic organisms in the lakes. The results showed that the lakes faced a threat hormone pollution though it was at lower doses and, thus, the ecological risk of EDCs should be considered in future environmental policies and decisions in China.

## 1. Introduction

Endocrine-disrupting compounds (EDCs), also known as environmental endocrine-disrupting chemicals, interfere with normal hormone levels in the body and are frequently detected in environments around the world [1,2,3]. EDCs can be roughly classified into three categories according to their basic structure and properties: fat-soluble organochlorine compounds, natural and synthetic estrogens and other substances with environmental hormone effects [3]. The awareness, research and prevention of environmental hormone pollution problems have begun to receive global attention. The pollution profile of environmental hormone-based pollutants in various environmental elements has been investigated in Japan. The Ministry of the Environment published the “Strategic Programs on Environmental Endocrine Disruptors 1998”, which lists 67 chemicals with environmental hormonal effects [4]. In the EU watchlist published in 2018 [5], a number of priority-controlled pollutants with environmental hormonal effects are listed, including the steroid estrogen 17α-EE_2_ and the natural estrogens E_1_ and E_2_ [6]. However, limited information is available regarding the distribution of EDCs in waterbodies of China.

EDCs in water mainly come from sewage treatment plants, livestock, agriculture and aquaculture waste, municipal waste and industrial activities [7]. These EDCs are not completely removed from sewage in traditional wastewater treatment plants [8]. EDCs can enter organisms via food, exposure, respiration, gills and the body surface of aquatic animals [9]. Although low in the environment, EDCs have strong hormonal effects and can cause endocrine disruption [10]. In aquatic environments, fish are subjected to EDCs that cause endocrine disruption, malformations and reproductive disorders, which in turn affect invertebrates, amphibians, reptiles, birds and mammals as they pass through the food chain [11,12]. The human endocrine system is highly sensitive to EDCs and these disruptors can interfere with metabolism, growth and development at different stages of life, particularly during embryonic and adolescent periods. During these stages, genetic reprogramming may be associated with impaired development and control of tissues and organs and these effects can even extend into adulthood [13]. EDCs may also lead to increased incidence of endocrine diseases and breast and prostate cancers [14]. Current data suggest that exposure to bisphenol analogues may have adverse effects on endocrine system dysfunction, such as tumors, neurotoxicity, reproductive toxicity and endocrine disruption [15]. China, the EU, the USA, Canada and other countries have enacted laws and regulations to restrict or ban the use of BPA [16]. The most common synthetic estrogen is 17α-EE_2_, which, due to the potential adverse effects of 17α-EE_2_ on human health, has been included in Japanese drinking water quality standards [17]. Synthetic estrogens also lead to hermaphroditism, reproductive disorders and population decline [18]. However, there are currently no clear laws and regulations in China to control estrogen contamination. Thus, EDCs in the environment have a potentially important impact on organisms, but few studies have investigated their occurrence and distribution.

Wuhan is an important city in the middle reaches of the Yangtze River in China. Major industries include optoelectronics, automotive manufacturing, steel manufacturing, new pharmaceuticals, bioengineering and new materials. There are 166 lakes in Wuhan, which have complex water bodies. Thus, the lakes in Wuhan face water pollution [19]. The completion and operation of these manufacturing industries and new projects might cause EDC pollution in waterbodies. Part of the global concern to date has focused on the occurrence of EDC contamination in the natural environment, mainly from industrial sources [20]. The industrial wastewater could contain a wide range of EDCs and bisphenol A (BPA); estrogen diethylstilbestrol and others could be detected in wastewater generated by manufacturing industries in particular [21]. However, few studies have investigated EDCs and their endocrine-disrupting effects in the lakes of Wuhan. In this study, 12 lakes in the exurban zone, suburban area and urban areas of Wuhan were selected for environmental hormone investigation from the perspective of population density. To our knowledge, this study is the first systematic investigation of the distribution of EDCs in surface waters of inland lakes. The spatial distribution characteristics of the emerging contaminants and the correlation between water quality parameters and environmental hormone content are elucidated. Finally, the potential ecological risk of these substances to the aquatic environment is assessed. Therefore, this study provides an assessment of the ecological risk of EDCs at lower doses and the present situation of hormone pollution threatening inland lakes in China and can thus be used as references of the ecological risk of EDCs in future environmental policies and decisions of China.

## 2. Materials and Methods

### 2.1. Study Area and Sample Collection

Wuhan is located in the eastern Jianghan Plain on the confluence of the Yangtze and Han River. It is in a subtropical temperate monsoon climate zone with abundant rainfall [22]. Wuhan has a total of 166 lakes, with a total area of 867 km^2^, accounting for 40% of the city’s 2117.6 km^2^ of water area. Twelve lakes in Wuhan, located on both sides of the Yangtze River, in exurban zone, suburban area and urban areas were selected for sampling. They included Julong Lake (JLL), Shanxishai Lake (SXS), Xiaomoshui Lake (XMSL), Longwanggou Lake (LWG), Longyang Lake (LYL), Huangjia Lake (HJL), Xiaoguanlian Lake (XGLL), Zhushan Lake (ZSL), Bei Lake (BL), Sanjiao Lake, Beitaizi Lake (BTZL) and Kuzhuhai Lake (KZL). Three of these lakes (HJL, ZSL, JLL) were found to have high levels of total EDCs and were used as example lakes to speculate on possible sources of pollution.

HJL has a water area of 811.8 ha and its main function is storage. The current water quality is class V and is moderately eutrophic. There are two outfalls into the lake, one drainage outfall and one water intake facility. There are 6 mixed inlets and 12 rainwater inlets into the lake. ZSL covers an area of 446 ha and its main function is water storage. There are 10 stormwater inlets and 1 drainage inlet along ZSL. The area of JLL is 95.8 ha. At present, the water quality is V class and eutrophic. The main function of JLL is aquaculture. There are no protective measures and water extraction facilities along the embankment of JLL. Rainwater and sewage directly flow into the lake.

**Figure 1:**
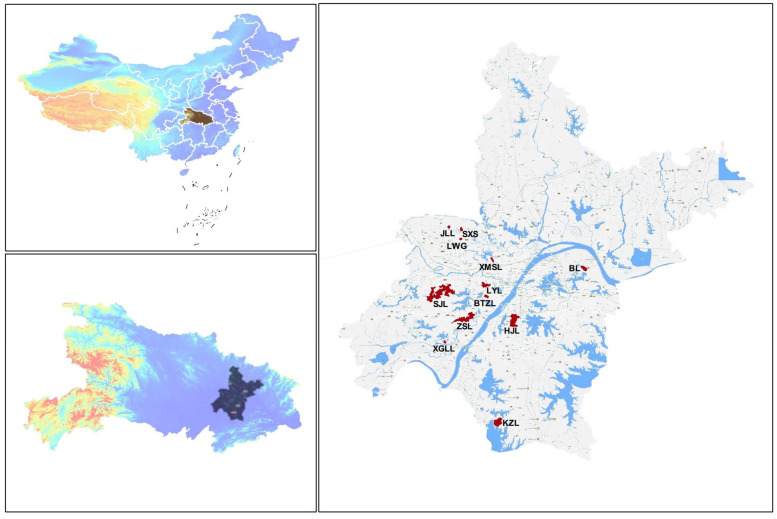
Map with the location of the sampling points in Wuhan Lakes.

Water samples were collected in March 2018 (Figure 1). As shown in Table S1 of the Supplementary Material, a total of 40 sites were adjusted according to the lake area. Mixed water samples were collected at a depth of 0.2 m below the surface. They were collected in stainless steel drums, stored in 4 L brown glass bottles and transferred to a 4 °C freezer until further processing 48 h later. Before collecting the sample, the bottles were pre-washed with distilled water and methanol and methanol was added immediately to inhibit microbial activity [23].

### 2.2. Sample Preparation

A modified extraction procedure was performed following a previously reported method [24]. The water samples were pumped through a 0.45 μm nitric acid fiber filter membrane to remove suspended particles in water. The pH was adjusted to 3 by 0.01 mol/L hydrochloric acid and sodium hydroxide. The HLB cartridges were pre-conditioned with 10 mL of high-performance liquid chromatography (HPLC) grade methanol followed by 10 mL of HPLC grade water and then 500 mL of filtered sample was passed through the cartridge at a flow rate of 5–8 mL/min. After extraction, the column was washed with 10 mL of 5% methanol, dried under vacuum at room temperature for half an hour and eluted with 6 mL of methanol. Then, the collected samples were evaporated under nitrogen flow. Finally, all eluents were reconstituted with 2 mL of methanol and filtered through a 0.22 μm filter membrane before instrumental analysis.

### 2.3. Analysis of EDCs by HPLC

Standard solutions and samples were analyzed by using an Agilent 1290 ultra-high-performance liquid chromatograph coupled to an Agilent 6460 triple quadrupole mass spectrometer. The electrospray ionization source was in negative and positive modes simultaneously. The analytes were separated on an Agilent Eclipse Plus C18 column (100 mm × 2.1 mm, 3.5 μm). The mobile phase was 1 mmol/L ammonium fluoride solution (A) and acetonitrile (B). The flow rate was 0.3 mL/min. The injection volume was 10 μL and the column temperature was 30 °C. The gradient elution conditions were as follows: 30% B from 0 min to 4 min; 30–90% B from 5 min to 14 min; then 70% B held for 5 min. Mobile phase gradient elution was performed and then held for 3 min. In multiple reaction monitoring acquisition mode, the mass spectrum parameters mainly include the precursor ion, product ion, collision energy and qualitative and quantitative ion equivalence (Table S2). The ion source temperature was 350 °C, the drying gas flow was 10 L/min, the heath gas flow rate was 11 L/min, the atomizer pressure was 3.1 × 10^5^ Pa and the capillary voltage was 3500 V. The target compounds were quantified by internal standard method. Ultrapure water blanks were analyzed for each analysis series and no target EDCs were detected. A spiked blank was prepared with every five samples for quality control. The recoveries of the target EDCs ranged from 73.94% to 108.7% with RSDs of 8.7% to 14.8%, which met the quality control requirements (Table S3).

### 2.4. Measurement of Water Quality Parameters

The temperature (T), pH, dissolved oxygen (DO), electrical conductivity (Pi), permanganate index (σ), nitrate nitrogen (NO_3_-N) and ammonia nitrogen (NH_3_-N) were measured by using a YSI Professional Plus Mi parameter meter (HACH, USA). Total phosphorus (TP) was measured by ammonium molybdate spectrophotometry and total nitrogen (TN) was determined by ultraviolet spectrophotometry [25].

### 2.5. Ecological Risk Assessment

The potential risks of EDCs in the surface water to aquatic organisms were assessed using the risk quotient (RQ) method according to Equation (1):

RQ = MEC/PNEC(1)

where MEC is the measured environmental concentration of the target compound and PNEC is the predicted no effect concentration of the target compound, which was calculated according to Equations (2) and (3):

PNEC acute = EC_50_(LC_50_)/1000 (2)

PNEC chronic = ChV/100 (3)

where EC_50_ (LC_50_) represents 50% effective (lethal) concentrations and ChV represents chronic toxic effects. EC_50_, LC_50_ and ChV were obtained from the ECOSAR model (US, EPA). The ECOSAR model is based on structural similarity, using the toxicity of compounds that have been previously evaluated in the aqueous environment as a reference and the procedure is widely used to predict the toxicity of various compounds in the aqueous environment [26]. The model selected for this study includes three trophic levels of aquatic ecosystems: fish, *Daphnia* and green algae (Table S4). RQ is divided into three risk levels. RQ < 0.1 (low risk), 0.1 < RQ < 1 (medium risk) and RQ > 1 (high risk). The quotient value is proportional to the risk [27].

### 2.6. Statistical Analysis

SPSS 13.0 for Windows (SPSS Inc., Chicago, IL, USA) was used for statistical analyses and Origin 8.5 (Origin Lab Corporation, Northampton, MA, USA) was used for data visualization in this study. Detrended correspondence analysis (DCA) and redundancy analysis (RDA) were performed by using Canoco 5. The results were presented by CanoDraw (Windows program) in the form of ordination diagrams. Spatial distribution of the sampling site was determined by ArcGIS software 10.2 (Redland, CA, USA).

## 3. Results and Discussion

### 3.1. Variance of EDC Content in 12 Lakes

**Figure 2:**
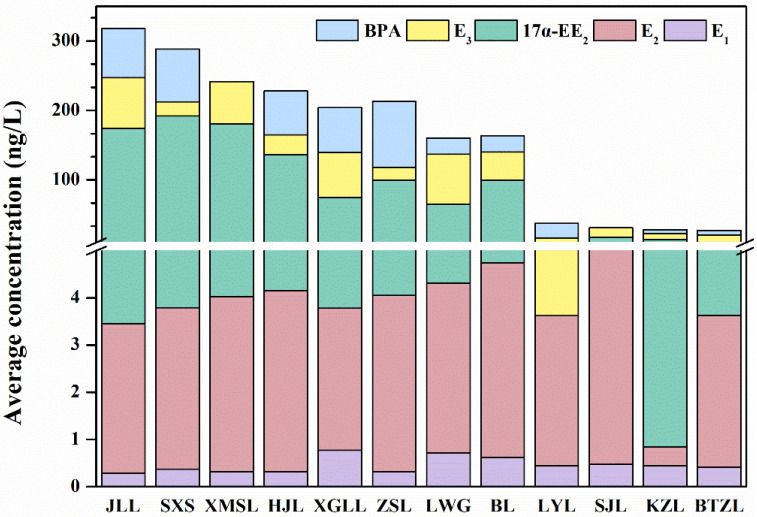
Concentrations of EDCs in the samples.

Considering the lack of data to support the detection rates, concentrations and impact of emerging pollutants in China, it is difficult for the government to develop regulations to control and manage pollutants that already persist in the environment. To date, no laws or orders have stated the upper limits for concentrations of pollutants that occur in wastewater discharges, drinking water or the environment [28]. In this study, five EDCs, namely, 17α-EE_2_ E_1_, β-E_2_, E_3_ and BPA were found in the surface waters of 12 lakes (Table S5). As shown in Figure 2, 17α-EE_2_ and BPA had high concentrations. E_3_, β-E_2_ and E_1_ had low concentrations. The maximum concentrations of 17α-EE_2_ and BPA were 188.2 and 95.19 ng/L, respectively, which were detected in SXS and ZSL. The content of 17α-EE_2_ ranged from 0 ng/L to 188.2 ng/L and the average concentration was 84.76 ng/L. The levels of BPA ranged from 0 ng/L to 95.19 ng/L with a mean concentration of 37.43 ng/L. The total concentrations of the three remaining endogenous estrogens (E_1_, E_2_ and E_3_) ranged from 0.29 ng/L to 73.11 ng/L (average concentration of 0.46, 3.35 and 35.69 ng/L, respectively). The detected concentrations of the target EDCs were at the ng/L level, which is a relatively low level in the environment as shown in Table S6. In most of the lakes, the content of EDCs detected was ordered as follows: 17α-EE_2_ > BPA > E_3_ > β-E_2_ > E_1_. Synthetic estrogens are more concentrated than natural estrogens [29]. As synthetic substances, 17α-EE_2_ and BPA were difficult to be degraded in the environment, which had higher concentration and cause cancers [30,31]. 

Previous studies detected different types of EDCs in water around the world, similar to the results of this study (Table 1). Even at concentrations between 10 ng/L and 100 ng/L, EDCs can cause irreversible damage to organisms [32]. The EDC concentrations in this study suggested that they might have detrimental effects on the growth and development of organisms.

### 3.2. Spatial Distribution of EDCs in 12 Lakes of Wuhan

**Figure 3:**
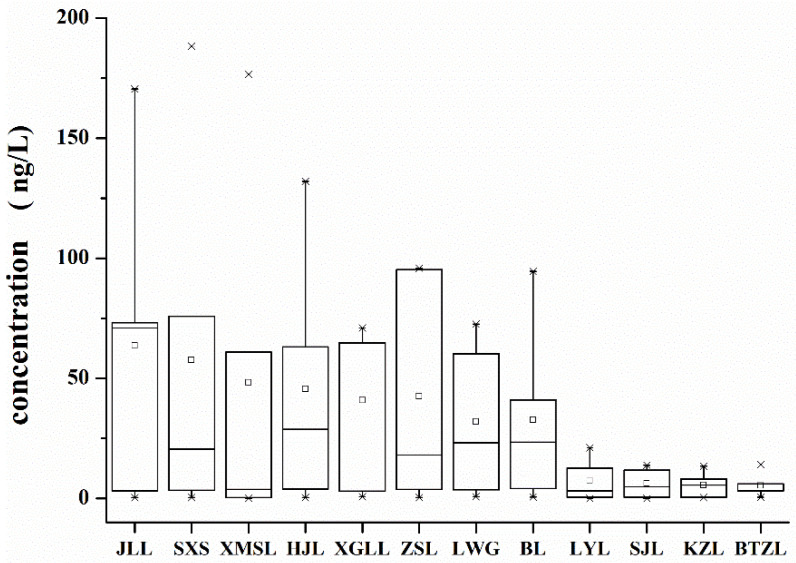
Boxplot of EDCs in 12 lakes. Discrete trends in EDCs measured at each sampling site in 12 lakes. The × reveals the minimum and maximum values of a data set.

The spatial distribution of EDCs in the Wuhan lakes was obvious and the total concentration of target compounds varied considerably between lakes in different geographical locations (Figure 3). The highest levels of EDCs were found in JLL, SXS and XMSL. The levels were 318.03 ng/L in JLL, 288.32 ng/L in SXS and 241.49 ng/L in XMSL. BTZL had the lowest total environmental hormone content at 26.83 ng/L, accounting for only 8.4% of the total EDC content of JLL. Apart from this, KZL, SJL and LYL had lower levels of target EDCs compared with the total environmental hormones of the 12 lakes (LYL 37.37 ng/L, SJL 30.83 ng/L and KZL 28.04 ng/L). In summary, the spatial distribution of environmental hormone pollution in water bodies showed variability.

**Figure 4:**
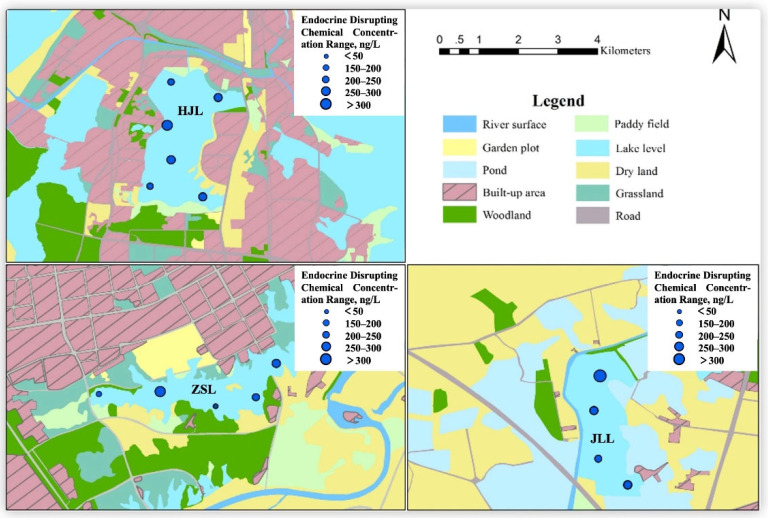
Distribution maps of EDCs in Wuhan Lakes.

On the basis of available information and surveys, three lakes (ZSL, HJL, JLL) in the suburban and urban areas of Wuhan were selected to analyze the possible cause of pollution by EDCs from different regions (Figure 4). The land use around the lakes and the amount of population are shown in Table 2. 

First, HJL is located in an urban area and has the largest proportion of floor space in the surrounding land use types, 37.21% (Table 2) including four universities, residential areas, markets and hospitals. The lake has 2 sewage draining exits, 6 mixed outfalls and 12 stormwater inlets. Thus, the main sources of pollution are domestic sewage and medical wastewater, which have a huge impact on the hormone content of the lake environment. EDCs can enter the water environment through direct domestic sewage discharge, sewage treatment plant effluent, percolation (e.g., septic tanks and landfills), stormwater runoff and accidental leakage [49]. The water treatment processing technology varies between different wastewater treatment plants and the various steps in the water treatment process can remove estrogenic activity to some extent, but they cannot effectively remove all EDCs. Similar to the previous study [50], EDCs are ubiquitous in the environment and have been found in drinking water in South Africa. Some of these EDCs are discharged into the aquatic environment through wastewater from households or hospitals, commercial or agricultural activities and industries and wastewater from sewage treatment plants that have not been properly treated.

Second, ZSL is located in a suburban area. According to the map, the area has the largest proportion of floor space by land use type at 31.58% (Table 2) and is dominated by a number of large factories and industrial areas, residential areas and a park. In recent years, along with the development of Wuhan’s industries, the lake has been filled in and reduced in size for the purpose of urban construction and renovation of the old city. The dramatic increase in the surrounding population, the discharge of domestic sewage and the deposition of waste in the vicinity inevitably added to the burden of the lake and affected its environment, leading to environmental pollution problems due to further deterioration of the water quality. In addition, building land accounted for the largest proportion, followed by agricultural land at 15.44%. The lake has 10 rainwater inlets and environmental hormones from agricultural production can enter the lake through surface runoff during rain and bring pollution.

Third, JLL is located in a remote suburban area. Its main function is aquaculture. The lake is surrounded by a large area of agricultural land, accounting for 44.86%; ponds at 32.01%; and construction areas at 0.12% (Table 2). There is no outfall and rainwater inlet into the lake, so agricultural production and aquaculture are the main pollution sources. Agricultural production and aquaculture were the main sources of EDCs in the aquatic environment and the presence of various environmental hormones was detected in different organs of fish [51].

In summary, the level of EDCs is related to the geographical location of the lake and the trend in concentration with geographical variation is exurban zone < suburban area < urban areas, which was similar to previous study [52]. The types and levels of EDC pollution can vary depending on the function of the lake and the different land uses surrounding it [53]. The main sources of pollution are sanitary sewage, wastewater from agricultural activities, industrial activities and waste from aquaculture fisheries. Similar findings have been made in previous studies [46,54,55,56,57]. Therefore, the distribution of EDCs in lakes has significant regional variability and is strongly influenced by human activities [40].

### 3.3. Correlations between EDCs and Water Quality Parameters

**Figure 5:**
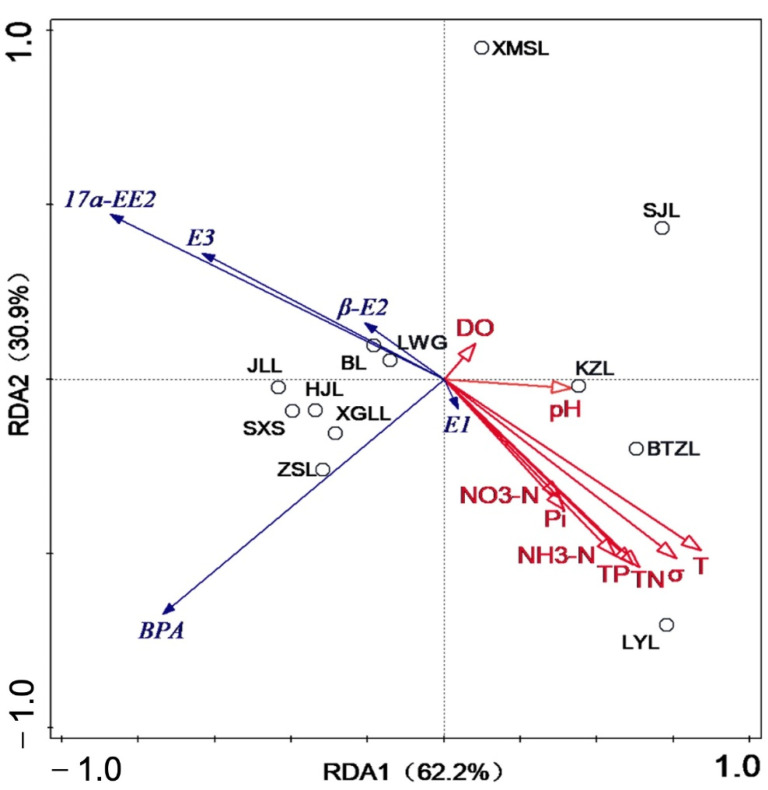
RDA analysis of the relations between EDCs and environmental factor contents.

DCA is widely used to analyze spatial variables or environmental risk associated with ecological factors [58]. To obtain correlations between target EDCs and measured water quality parameters, a DCA was performed on all water samples. The water quality parameters (e.g., T, TN, TP, NH_3_-N, NO_3_-N, pH value, DO, Pi and σ) for each sampling site are listed in Table S7. The results showed that the length of the first axis was 0.8 (Table S8). On the basis of this result, RDA was used to analyze the effects of community structure and environmental factors [59]. In Figure 5, the longer the ray, the greater the influence of the environmental factor. A positive correlation is indicated by an acute angle between two environmental factors, while negative correlation is indicated by an obtuse angle. The results showed that the first axis of the RDA ordination plot showed a correlation coefficient of 0.8 and a variance of 62.2% (Table S9). There was a positive correlation between E1 and all selected environmental variables except DO. The EDCs were eliminated with relatively high rates under aerobic conditions in a river water system and biotransformation would decrease under reduced oxygen conditions [42]. It usually is thought that DO is negatively correlated with concentrations of some EDCs and affects the environment’s fate and distribution of EDCs in terms of redox, biodegradation and biotransformation [54]. Moreover, Le Thi Minh found a positive correlation between organic and nitrogen compound contaminated surface water and some environmental hormone pollution [33]. DO and pH were almost all negatively correlated with the five hormones, while T, TP, TN, NH_3_-N, NO_3_-N and Pi were strongly negatively correlated with E_3_, 17α-EE_2_ and E_2_. After entering the aqueous environment, EDCs may undergo a series of transport and transformation processes such as photolysis, microbial degradation and adsorption. These processes may be influenced by water quality parameters such as DO, pH and T, which can enhance or reduce the degradation of the compounds and affect their chemical stability and environmental behavior [57]. At present, the mechanism behind this aspect remains to be elucidated. Thus, research on this aspect should be carried out in the future to determine the correlation between water body parameters of different lakes or regions.

### 3.4. Ecological Risk Assessment

**Figure 6:**
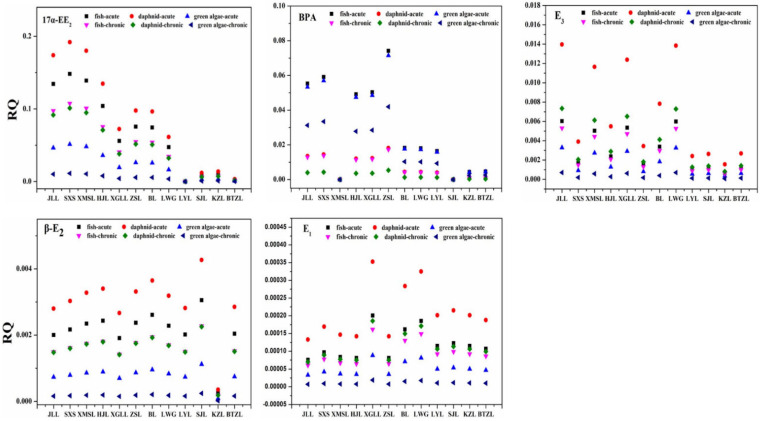
Risk quotients (RQs) calculated for detected compounds in each sampling site.

As shown in Figure 6, the RQ values of target EDCs in the 12 lakes decreased in the following order: 17α-EE_2_ > BPA > E_3_ > E_2_ > E_1_. The RQ values of 17α-EE_2_ exceeded 0.1 at some of the sampling sites, while those of the other four EDCs were lower than 0.1, which was the least risk state for all levels of the tested organisms. Among the five EDCs, 17α-EE_2_ had the highest risk factor, especially for acute risk to *Daphnia*. 17α-EE_2_ was considered to be the most hazardous chemical among the target EDCs with a median RQ of 8.94 in a potential ecological risk assessment of residual endocrine disruptors in selected wastewater treatment plants in Guangdong [41]. The risk value of SXS exposure was the highest in 12 lakes, reaching 0.19. Therefore, *Daphnia* might be at a moderate risk of exposure to estrogenic EDCs. Figure 6 also shows that fish are the second most sensitive of the three tested species, while green algae are the least sensitive. 

The second most harmful EDCs to aquatic organisms was BPA, with an RQ close to 0.08 in ZSL, but still in the lowest risk category. Fish had a slightly higher acute toxicity RQ than the other organisms. The RQ of BPA to some aquatic organisms in Luoma Lake and Taihu Lake was lower than 0.1 [16], which was similar to this study. The second most sensitive organism was green algae, followed by *Daphnia*. The RQs of these organisms were in the low risk state. 

For E_1_ and β-E_2_, *Daphnia* had the highest acute risk value, but both compounds were in the lowest risk state. Fish was the second most sensitive organism to E_1_ and β-E_2_, while green algae had the lowest sensitivity, which was similar to previous research [60]. For E_3_, *Daphnia* had the highest acute risk value, but was still lower than 0.1. In general, the total potential toxic risk of the five detected EDCs in the surface water of 12 lakes in Wuhan was low and presented moderate risk to aquatic organisms. Although the levels of EDCs detected so far are very low and environmental pollutants need to accumulate to a certain amount before they become toxic, people need to be constantly alert. In humans or animals, EDCs can be present in the blood [55,61], accumulate and travel with the bloodstream to target cells, bind to specific receptors on the cell membrane and compete with the organism’s own hormones for receptors (hormone binding sites), thus affecting their normal physiological activities [62]. The relevant government departments should prioritize the selection of potentially dangerous environmental hormones as targets for control, set standards and implement control and emission restrictions.

## 4. Conclusions

Environmental endocrine-disrupting chemicals, such as 17α-EE_2_, E_1_, E_2_, E_3_ and BPA, were emerging contaminants in the surface waters of the sampling lakes. In this study, E_1_, E_2_ and E_3_ were found in all sampling sites. Among the detected EDCs, BPA and 17α-EE_2_ were the predominant compounds detected in surface water. All the EDCs had relatively high concentrations in the bay of the lake near densely populated areas. Sanitary sewage, industrial effluents, agricultural wastewater and aquaculture sewage were identified as the major sources of EDCs. The basic water quality parameters (pH, T and DO) played important roles in determining the distribution, fate and behavior of EDCs in surface water. The environmental risk of each compound was assessed by RQ ranking, with the target compounds all having a low or medium risk status. This study provided a comprehensive understanding of the current state of EDC pollution of lakes in Wuhan and could be used as a reference for the effective control of environmental pollution risks in inland lakes. What’s more, through the investigation and risk assessment of EDC pollution in Wuhan lakes, the general public should be fully aware of the harmful effects of EDC pollution. EDCs are mainly harmful to the reproductive functions of humans and animals, seriously threatening their survival and reproduction. In the future, China should strengthen basic research on EDC pollution, enhance environmental management and publication of environmental laws and improve water purification measures in sewage treatment plants in order to curb EDC pollution. In addition, some legislative measures should be proposed to develop laws and regulations on EDCs and to strengthen enforcement to control EDCs.

## Figures and Tables

**Table 1:** Comparison of EDC concentrations in the surface waters around the world.

Area	EDCs	Concentration (ng/L)	References
Vietnam, Sai Gon and Dong Nai river basin	Nonylphenol	5.9–235	[33]
Southwest Germany, river Rhine	17α-EE_2_	<1.5	[34]
South Africa, south of Johannesburg	E_1_	0.90–4.43	[35]
Morocco, Bouregreg River	Nonylphenol	11–200	[36]
Morocco, Bouregreg river	E_1_	5–277	[36]
Morocco, Bouregreg river	E_2_	21–200	[36]
Brazil, five full-scale wastewater treatment plants	17β-E_2_	ND–776	[37]
Brazil, Sinos River basin	BPA	ND–517	[38]
China, Lhasa River Basin	E_1_	ND–3.9	[39]
China, Lhasa River Basin	BPA	ND–433	[39]
China, Honghu Lake	17α-EE_2_	ND–33.28	[40]
China, 38 wastewater treatment plants	β-E_2_	ND–62.92	[41]
China, Chaobai watershed	E_3_	ND–23.51	[42]
China, Jiulong river and estuary	E_3_	ND–118	[43]
Dan-shui River	E_3_	ND–73.5	[44]
China, three rivers in Tianjin	17α-EE_2_	1.55–24.40	[45]
China, Taihu Lake	BPA	ND–112	[46]
China, Taihu Lake	4-nonylphenol	ND–324	[46]
China, Xiangshui River and Heng River	17β-boldenone	ND–0.91	[47]
China, Yangtze River (Nanjing section)	BPA	1.7–563	[48]

**Table 2:** Percentage of each type of land use and the amount of population in the three lake areas.

	JLL		HJL		ZSL	
Terrain Category	Class Size	Proportion	Class Size	Proportion	Class Size	Proportion
road	0.18	4.45	7.67	6.17	3.58	5.67
farmland	1.77	44.86	9.32	7.50	9.76	15.44
river	0.05	1.23	1.04	0.84	2.09	3.30
lake	0.36	9.14	38.26	30.80	7.36	11.65
built-up area	0.12	3.06	46.23	37.21	19.96	31.58
pit-pond	1.26	32.01	1.10	0.89	1.93	3.05
woodland	0.19	4.78	10.63	8.56	7.92	12.52
paddy field	0.02	0.40	3.38	2.72	4.59	7.26
garden plot	0.00	0.06	6.57	5.29	6.03	9.53
population	2019		106,087		29,824	

## Data Availability

Not applicable.

## Supplementary Materials

The following supporting information can be downloaded at: https://www.mdpi.com/article/10.3390/toxics10020093/s1, Table S1: Basic information for each sampling site of the lakes; Table S2: Mass spectrometric parameters and retention time; Table S3: The method validation parameters of EDCs in surface water samples; Table S4: ECOSAR data of target environmental hormone and PNEC calculation; Table S5: The main detected EDCs and its information; Table S6: Values of EDCs in the samples; Table S7: Key environmental parameters of sampling water from twelve lakes in Wuhan City; Table S8: Summary of the forward selection procedure in the redundancy analysis (RDA) performing the data from the water samples; Table S9: Eigen values for redundancy analysis (RDA) and correlation coefficients between environmental factors and RDA ordination axes.
