# Supplementary material for: Pollution Characteristics and Risk Prediction of Endocrine Disruptors in Lakes of Wuhan
Source: Toxics. 2022 Feb 18;10(2):93. doi: 10.3390/toxics10020093 (PMC8880694; doi:10.3390/toxics10020093)
Supplement: Supplementary file 1 [file toxics-10-00093-s001.zip › toxics-1556221-supplementary.pdf]

toxics MDPI

Supplementary Materials: Pollution Characteristics and Risk Prediction of Endocrine Disruptors in Lakes of Wuhan

Yurui Zhang, Jun Cao, Tan Ke, Yue Tao, Wanyin Wu, Panpan Wang, Min Zhou and Lanzhou Chen

Table S1. Basic information for each sampling site of the lakes.

Sites	administrative region	Location	area (hm²)	Sampling number
JLL	Dongxihu District	E114.07, N30.75	95.8	4
SXS	Dongxihu District	E114.14, N30.75	23.3	1
XMSL	Dongxihu District	E114.22, N30.66	15.8	1
LWG	Dongxihu District	E114.13, N30.73	18.2	1
LYL	Dongxihu District	E114.19, N30.56	168	3
HJL	Hanyang District	E114.29, N30.44	811.8	6
XGLL	Caidian District	E114.06, N30.37	29	3
ZSL	Hannan District	E114.13, N30.45	446	5
BL	Jianghan District	E114.52, N30.61	9.4	2
SJL	Jingjikaifa District	E114.18,N30.53	239.1	4
BTZL	Caidian District	E114.20, N30.52	52.4	3
KZL	Jiangxia District	E114.23, N30.03	725.1	7

Table S2. Mass spectrometric parameters and retention time.

Compound	Retention time, min	Precursor ion, m/z	Product ion m/z	Collision Energy, V	ESI Mode
E_3	1.575	287.2	171*/143	38/38	ESI-
E_1	5.622	269.1	145.1*/183	33/38	ESI-
β-E_2	5.150	271.2	145.1*/183	40/40	ESI-
17α-EE_2	5.468	295.2	260.9*/183	17/33	ESI-
BPA	4.970	227.1	212.1*/133.1	16/24	ESI-

Table S3. The method validation parameters of EDCs in surface water samples.

Analyte	R^2	Recoveries (spiked at 50 ng L^{-1})	RSD[a] (%)	LOD ng/L
E_1	0.999	86.2	8.7	0.2
E_2	0.996	86.67	11.6	1.1
E_3	0.998	108.7	10.4	1.2
17α-EE2	0.999	73.94	14.8	1.8
BPA	0.998	74.23	9.3	1.0

Table S4. ECOSAR data of target environmental hormone and PNEC calculation.

Compound	Class	Time	LC50/EC50 (mg/L)	Assessment factor	PNEC （ng/L）
E_1	fish	96	3.834	1000	3834
	daphnid	48	2.184	1000	2184
	green algae	96	8.74	1000	8740
	fish	chv	0.477	100	4770
	daphnid	chv	0.415	100	4150
	green algae	chv	4.032	100	40320
BPA	fish	96	1.284	1000	1284
	daphnid	48	5.237	1000	5237
	green algae	96	1.331	1000	1331
	fish	chv	0.55	100	5500
	daphnid	chv	1.773	100	17730
	green algae	chv	0.227	100	2270
E_3	fish	96	12.115	1000	12115
	daphnid	48	5.235	1000	5235
	green algae	96	22.252	1000	22252
	fish	chv	1.377	100	13770
	daphnid	chv	0.995	100	9950
	green algae	chv	10.347	100	103470
17α-EE_2	fish	96	1.269	1000	1269
	daphnid	48	0.98	1000	980
	green algae	96	3.671	1000	3671
	fish	chv	0.175	100	1750
	daphnid	chv	0.186	100	1860
	green algae	chv	1.679	100	16790
B- E_2	fish	96	1.578	1000	1578
	daphnid	48	1.129	1000	1129
	green algae	96	4.299	1000	4299
	fish	chv	0.212	100	2120
	daphnid	chv	0.214	100	2140
	green algae	chv	1.97	100	19700

Table S5. The main detected EDCs and its information.

Compound	Formula	Molecular Mass, g/mol	Chemical structure	CAS number
E_1	$C_{18}H_{22}O_2$	270.37	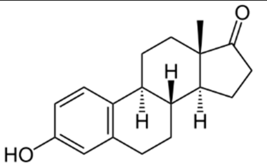	53-16-7
E_2	$C_{18}H_{24}O_2$	272.38	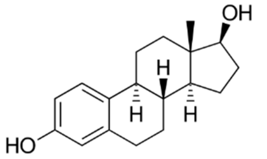	50-28-2
E_3	$C_{18}H_{24}O_3$	288.38	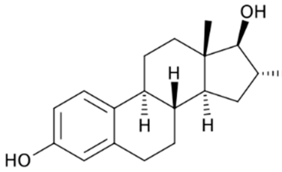	50-27-1
17α-EE_2	$C_{20}H_{24}O_2$	296.40	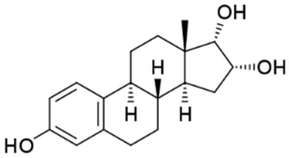	57-62-6
BPA	$C_{15}H_{16}O_2$	228.29	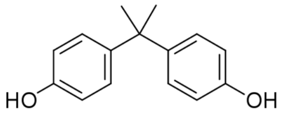	80-05-7

Table S6. Values of EDCs in the samples.

Analyte	E1 ng/L	BPA ng/L	E3 ng/L	17a-EE2 ng/L	β-E2 ng/L
JLL	0.29	71	73.11	170.47	3.16
SXS	0.37	75.86	20.47	188.2	3.42
XMSL	0.32	0	60.96	176.51	3.7
HJL	0.31	63.13	28.75	132.07	3.84
XGLL	0.77	64.63	64.86	70.95	3.01
ZSL	0.31	95.19	18.03	95.86	3.74
BL	0.62	23.46	40.98	94.54	4.12
LWG	0.71	23.13	72.51	60.18	3.6
LYL	0.44	21.14	12.61	0	3.18
SJL	0.7	0	13.78	11.76	4.82
KZL	0.44	5.58	8.15	13.47	0.4
BTZL	0.41	6	14.05	3.15	3.22

Table S7. Key environmental parameters of sampling water from twelve lakes in Wuhan City.

Sampling site	T (°C)	pH	DO (mg/L)	σ mg/L	NH3-N mg/L	TP mg/L	TN mg/L	NO3-N mg/L	Pi μS/cm
JLL	20.60	7.66	8.20	3.97	0.44	0.09	1.20	0.34	2.57
SXS	21.20	7.46	7.80	6.80	1.05	0.27	2.59	0.90	2.55
XMSL	20.18	7.53	8.20	3.40	0.56	0.17	1.60	0.49	2.55
HJL	25.76	8.53	10.27	5.29	0.48	0.14	0.92	0.09	2.56
XGLL	23.13	7.91	8.88	5.00	0.65	0.16	1.58	0.39	2.54
ZSL	22.95	8.59	11.92	4.58	0.48	0.14	1.25	0.14	2.48
BL	19.23	7.51	7.93	3.98	0.45	0.24	1.28	0.32	2.48
LWG	23.40	7.47	7.68	7.13	0.66	0.47	1.76	0.33	2.59
LYL	28.68	7.03	4.18	8.27	12.27	0.98	15.92	1.38	2.86
SJL	25.95	8.60	9.66	6.25	1.63	0.20	2.54	0.16	2.59
KZL	26.68	9.03	13.49	6.67	0.32	0.13	0.83	1.03	2.40
BTZL	25.40	9.83	12.92	9.76	0.71	0.26	2.75	0.10	2.51

Table S8. Summary of the forward selection procedure in the redundancy analysis (RDA) performing the data from the water samples.

**Environmental variable**	**Abbreviation**	**λ (%) [a]**	**p [b]**	**F**
Water temperature	T	35.6	0.008	5.5
Electrical conductivity	Pi	3.9	0.582	0.5
Permanganate index	σ	4.4	0.4	0.4
pH	pH	7.5	0.012	1.2
Dissolved oxygen	DO	10	0.003	7.7
Ammonia nitrogen	NH_3-N	0.8	0.942	0.1
Total phosphorus	TP	16.5	0.218	2.3
Total nitrogen	TN	6.1	0.37	0.9
Nitrate nitrogen	NO_3-N	1.9	0.74	0.2

λ (%)[a]: the variation explained by the selected variables;.p [b]: significance of explained variation;.The bold number means the significant level at $p < 0.01$; Statistical significance was tested using Monte Carlo permutation test with 499 permutations.

Table S9. Eigen values for RDA and correlation coefficients between environmental factors and RDA ordination axes.

**Axes**	**1**	**2**
Eigenvalues	0.622	0.309
Species-environment correlations	0.461	0.815
of species data	8.39	2.50
of species-environment relation	62.19	93.13
